# Does Oral Apigenin Have Real Potential for a Therapeutic Effect in the Context of Human Gastrointestinal and Other Cancers?

**DOI:** 10.3389/fphar.2021.681477

**Published:** 2021-05-18

**Authors:** Eva F. DeRango-Adem, Jonathan Blay

**Affiliations:** ^1^ School of Pharmacy, University of Waterloo, Waterloo, ON, Canada; ^2^ Department of Pathology, Dalhousie University, Halifax, NS, Canada

**Keywords:** apigenin, pharmacokinetics, pharmacodynamics, colorectal carcinoma, cancer, natural health products (NHPs), dietary contituents, therapeutics

## Abstract

Apigenin (4′, 5, 7-trihydroxyflavone) is a plant flavone that has been found to have various actions against cancer cells. We evaluated available evidence to determine whether it is feasible for apigenin to have such effects in human patients.

Apigenin taken orally is systemically absorbed and recirculated by enterohepatic and local intestinal pathways. Its bioavailability is in the region of 30%. Once absorbed from the oral route it reaches maximal circulating concentration (C_max_) after a time (T_max_) of 0.5–2.5h, with an elimination half-life (T^1^/_2_) averaging 2.52 ± 0.56h.

Using a circulating concentration for efficacy of 1–5μmol/L as the target, we evaluated data from both human and rodent pharmacokinetic studies to determine if a therapeutic concentration would be feasible. We find that oral intake of dietary materials would require heroic ingestion amounts and is not feasible. However, use of supplements of semi-purified apigenin in capsule form could reach target blood levels using amounts that are within the range currently acceptable for other supplements and medications. Modified formulations or parenteral injection are suitable but may not be necessary.

Further work with direct studies of pharmacokinetics and clinical outcomes are necessary to fully evaluate whether apigenin will contribute to a useful clinical strategy, but given emerging evidence that it may interact beneficially with chemotherapeutic drugs, this is worthy of emphasis. In addition, more effective access to intestinal tissues from the oral route raises the possibility that apigenin may be of particular relevance to gastrointestinal disorders including colorectal cancer.

## Introduction

Natural products have long been appreciated for their potential as molecular sources for novel chemotherapeutic and chemopreventive agents(Cassady et al., 1990). Among the diverse possibilities, flavonoids have shown particular promise as either natural molecules in the diet, or lead compounds for drug development (Kale et al., 2008; Androutsopoulos et al., 2010; Batra and Sharma, 2013). Among the flavonoids, one that has captured the attention of researchers such as ourselves has been apigenin, a flavone that is substituted by hydroxy groups at positions 4′, 5 and 7(National Center for Biotechnology Information, 2021). This plant-derived molecule has been of clinical interest since the 1950s, following observations that it is able to modulate histamine release and is bronchodilatory(Hava and Janku, 1958; Spicak and Subrt, 1958). However more recently, we and others have drawn attention to the possibility that apigenin may have useful properties against cancer(Lefort and Blay, 2013; Shankar et al., 2017; Yan et al., 2017). In particular, we have noted that apigenin’s action at the cellular level is distinct from that of closely related flavones such as kaempferol and genistein(Lefort and Blay, 2011; Lefort et al., 2020).

Our research has focused particularly on gastrointestinal cancers(Lefort and Blay, 2013). However, apigenin has potentially advantageous effects on cellular processes in many solid cancers, including those of the breast, prostate, lung and lymphoma, and leukemias (Lefort and Blay, 2013), supporting contentions that it may have a general benefit in managing different neoplasias (Shankar et al., 2017; Yan et al., 2017).

Among the challenges in taking advantage of such molecules in their natural context or an extracted form is their capacity to reach biologically active concentrations at the target site and their selectivity of action. Together these two considerations define the potential efficacy as a therapeutic or adjunct, and side effect profile. Although many effects can be demonstrated *ex vivo* or in preclinical models, the question of whether natural product molecules are clinically relevant *via* the ingestion route, or whether clinically important activity can only be attained with additional drug-delivery technologies or chemical modification, is often not clear.

For the oral route, there is the question of whether clinically relevant concentrations of natural product molecules can be achieved by simple dietary manipulation, or whether artificial supplements are necessary to reach the required dosing level. When addressing cancer, we face two different situations in that accessing most cancers depends on the phases of absorption and systemic distribution after oral ingestion of the molecule of interest. However for gastrointestinal cancers, which are our primary interest, dietary apigenin has relatively direct access to neoplastic tissue that is positioned close to the intestinal lumen, particularly in early stage disease.

In this article, we review the currently available data on pharmacokinetics and pharmacodynamics of apigenin, and place these data in the context of the apigenin’s potential for meaningful intervention in gastrointestinal and other oncopathologies. We will consider apigenin’s physical and physiological properties, chemical properties (e.g., solubility), the comparative evidence for *in vitro* and *in vivo* therapeutic effects, and pharmacokinetic properties (absorption, distribution, metabolism, elimination).

## METHODS

### Article Sourcing and Data Extraction

A database of 1,447 literature sources within the laboratory relating to flavonoid action in the gastrointestinal tract was used as the initial resource and to define best search parameters to capture all relevant articles relating to the pharmacokinetic and pharmacodynamic profile of apigenin.

To capture all articles relevant to the pharmacokinetics of apigenin, a broad search strategy using those two terms was used on PubMed, considering only articles written in English or available as an English translation. This yielded (as of September 2020) an additional 314 articles which were manually curated together with those already available to provide the basis for this review. Selection was for relevance to the questions posed, informed by the perspectives of the authors. We considered i) wide information from a variety of analytical and preclinical models, ii) comparisons between oral and other administration routes, and iii) all information relevant to absorption, distribution, metabolism and excretion (ADME; the four parts of pharmacokinetics or how the body handles a bioactive molecule).

To capture cellular mechanistic and pharmacodynamic data with particular attention to gastrointestinal cancers, the following search strategies were used in the same framework:

1. apigenin AND (vill* OR mucosa OR “lamina propria” OR mesentary OR capillar* OR lympat*)
2. apigenin AND (“plasma membrane” OR endosom* OR nucle* OR transporter*)
3. apigenin AND (conversion OR deglycos* OR hydroxy* OR hydroph*)
4. apigenin AND (HT-29 OR HT29 OR HCT-116 OR HCT116 OR HRT-18 OR HRT18 OR Caco-2 OR Caco2 OR T84 OR SW480 OR SW620 OR Colo-320 OR Colo320)
5. apigenin AND (cancer OR carcino*) AND (gastrointes* OR colo*)

Ths enabled interpretation of pharmacokinetic data with particular emphasis on intestinal cellular action and gastrointestinal oncopathologies, as is the focus of this review.

### String Interactome Mapping

STRING is a web-based database of known and predicted protein-protein interactions that allows protein-protein interaction mapping based on both direct (physical) and indirect (functional) associations (Szklarczyk et al., 2019). Potential protein interactions involving apigenin and the cellular regulators identified in the pharmacodynamic review of this project were subjected to STRING analysis using *Homo sapiens* as the model organism. Proteins analyzed included APC, p34 (CDK2), p21 (CDKN1A), CDK1, cyclin B1, PARP, Bcl-XL, Mcl1, Stat3, Bax, Cytochrome C, Caspase-3, Caspase-8, Caspase-9, CHOP (DDIT3), DR5 (TNFRSF10B), Nag1 (GDF15), p53, p21, Erk (p38, MAPK1), Beta-Catenin 1 (CTNNB1), Snail, NFKB1, AKT1, C-myc, cyclin D1, PKM2, PTBP1, DPP4, ADA, and CK2 (CSNK2A1). These proteins are represented as nodes on the STRING map, with connections ranked as specified in the figure legend.

## CURRENT KNOWLEDGE AND ANALYSIS

### Structure of Apigenin and its Relationship With Other Plant Flavones

**FIGURE 1:**
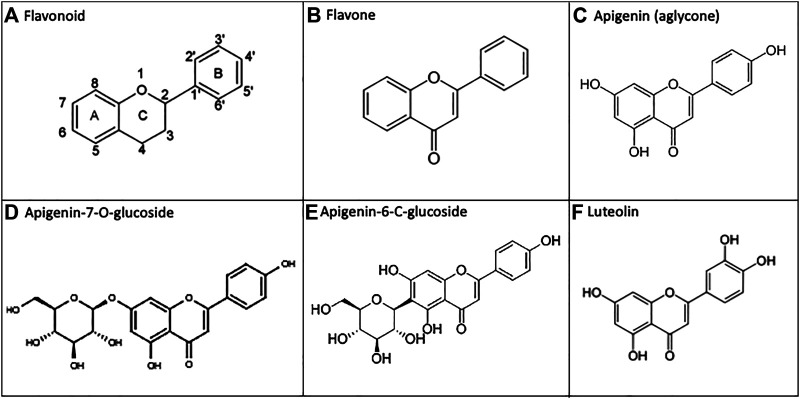
Apigenin and related chemical structures. **(A)** General flavonoid structure as 15-carbon molecules containing two phenyl groups and a heterocyclic carbon ring. **(B)** The flavone sub-class of flavonoids have a C2-C3 double bond, unsubstituted C3 carbon, and a C4 ketone oxidization. **(C)** Apigenin is a 4′, 5, 7-trihydroxyflavone. **(D, E)** In nature, apigenin is commonly found as a 7-O-glucoside, 6-C-glucoside or 8-C-glucoside, which is enzymatically metabolized to free apigenin prior to intestinal absorption. **F)** Apigenin undergoes phase I metabolism *via* CYP1A2, and to a lesser extent CYP3A4, generating the 3′-hydroxylated product luteolin.

Flavonoids are polyphenolic natural products derived from plants and present in high amounts within leaves, flowers, and fruits(Hostetler et al., 2017). These diverse chemical compounds serve as native signaling mediators, allow UV protection, or enable pesticidal control within multiple plant phyla(Hostetler et al., 2017; Nunes et al., 2018). When plants are consumed by animals, including humans, some of these molecules also exhibit anti-inflammatory and antioxidant properties which are believed to translate to health benefits. However, the low bioavailability of flavonoids constitutes a barrier to their efficacy via the oral route (Thilakarathna and Rupasinghe, 2013). Flavones are a sub-class of flavonoids, having a C2-C3 double bond, unsubstituted C3 carbon, and ketone oxidization at C4 (Panche et al., 2016) (Figure 1).

We have noted particular health potential for apigenin–a plant flavone found in high concentrations within parsley, chamomile, celery, spinach, artichokes and oregano(Shankar et al., 2017). Apigenin is a 4′, 5, 7-trihydroxyflavone (National Center for Biotechnology Information, 2021), and it is the free hydroxyl groups present on the A/B rings of apigenin that are responsible for the antioxidant effects of this flavone(Singh et al., 2014). In natural sources, apigenin is commonly found as an 7-O-glucoside, 6-C-glucoside, or 8-C-glucoside (14, 18) (Figure 1). After ingestion of the plant material, these glucosides are enzymatically metabolized *in vivo* into free apigenin (*i.e.,* the aglycone form) and subsequently absorbed(Hostetler et al., 2013; Hostetler et al., 2017). Luteolin is another plant flavonoid, and it is also an apigenin metabolite–produced as a result of phase I cytochrome P450 (CYP) enzyme hydroxylation *in vivo* (Hostetler et al., 2017) (Figure 1).

### Physico-Chemical Properties of Apigenin and Their Effect on Uptake

Flavonoids as a sub-class are poorly bioavailable from the diet due to their low water solubility, chemical instability, and rapid metabolism in the body (Leonarduzzi et al., 2009). Apigenin itself is however lipophilic and a weak acid, and therefore will be most permeable to cell membranes in the unionized form within acidic environments, allowing it to be better absorbed in lower pH environments along the gastrointestinal tract, including the stomach (Leonarduzzi et al., 2009). In the pH range of the intestinal tract, hydrophobic apigenin is still able to permeate lipid membranes–enabling absorption along the full length of the intestines, but does so most effectively in the duodenum (Tang et al., 2017).

Apigenin is a Class II molecule under the Biopharmaceutics Classification System (BCS) (Tsume et al., 2014), exhibiting low water solubility and high permeability (Leonarduzzi et al., 2009). Water solubility increases with ionization in more basic environments. The maximal aqueous solubility of apigenin occurs at a pH of 7.5, up to a concentration of 0.183mg/ml (National Center for Biotechnology Information, 2021).

### Overall Uptake of Apigenin From the Diet

Observational studies looking at consumption of apigenin within whole foods in the human diet indicate daily intake between 0.45 and 1.17mg–varying between age and demographic(Meyer et al., 2006; Somerset and Johannot, 2008). Meyer and colleagues studied the bioavailability of apiin (apigenin-7-O-apiosylglucoside) following a single bolus of parsley–a food source with an exceedingly high concentration of apigenin (Meyer et al., 2006). Blood and urine samples from 11 German adults (ages 23–41) were taken following a meal consisting of 2g parsley/kg body weight–which was equivalent to ∼17mg of apigenin (Meyer et al., 2006). Plasma concentrations of apigenin ranged from 28–337nmol/L at 6–10h after consumption, and fell below detection at 28h (Meyer et al., 2006). A total of 0.039mg (±0.03) of apigenin was recovered over 24h from urine samples (Meyer et al., 2006). These low plasma and urine concentrations are expected based on some of the pharmacokinetic parameters that will be discussed below - such as rapid metabolism, excretion of unabsorbed apigenin in non-urinary pathways, or hydrolysis by colonic microflora (Meyer et al., 2006).

### Absorption of Apigenin in the Gastrointestinal Tract

**FIGURE 2:**
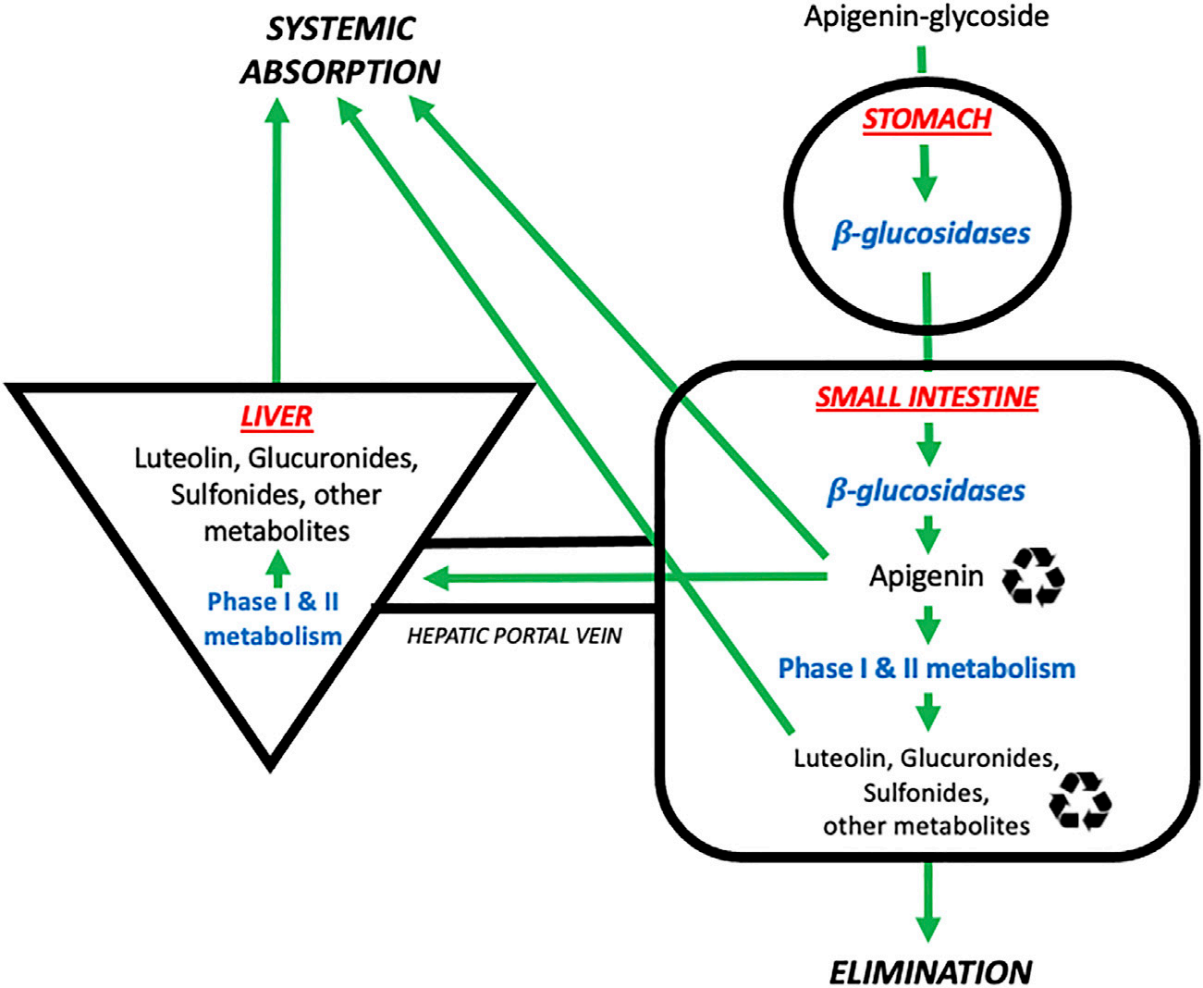
The absorption, distribution, metabolism, and elimination of apigenin. Apigenin is present in the diet in glycosylated forms found in nature (e.g., 7-O-glucoside, 6-C-glucoside or 8-C-glucoside). These glycosides are then metabolized by *β*-glucosidases in the stomach and small intestines to generate free apigenin (i.e., the aglycone form). Free apigenin can be directly absorbed systemically, or undergo downstream phase I and II metabolism in the small intestines and liver to generate hydroxylated metabolites such as luteolin, or glucuronidated and sulfonated metabolites. These metabolites enter four possible pathways: i) direct systemic absorption, ii) elimination (mostly via the urine, to a lesser extent the feces), iii) local enteric recycling, or iv) enterohepatic recycling via the bile. 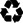
Denotes that molecules are subject to recycling through enteric and/or enterohepatic routes. Image adapted from (Thilakarathna and Rupasinghe, 2013).

A schematic summary of the physiological processes leading to the absorption, distribution, metabolism, and elimination of apigenin is shown in Figure 2. As mentioned above, apigenin is ingested in a glycosylated form. These apigenin glucosides must be metabolized to the aglycone form by enzymes in the stomach and/or small intestines–such as epithelial *β*-glucosidases–or within endogenous colon microflora, prior to systemic absorption (Srinivas, 2015). *Eubacterium ramulus* and *Bacteroides distasonis* have been identified as major bacterial species essential to the biotransformation of 7-glycosides into aglyconated apigenin (Hanske et al., 2009). *Ex vivo* hydrolytic deglycosylation studies of apigenin and luteolin into their respective aglycone forms using rat models suggest variability in deglycosylation rate according to location within the gastrointestinal tract (Lu et al., 2010).

Oral administration of extracts of *Chrysanthemum morifolium,* which contain high levels of apigenin in its 7-O-β-D-glucosidic form, has been studied in rats (Lu et al., 2010). There is substantial and fairly rapid early deglycosylation after ingestion in the stomach and upper intestine, with absorption beginning in the duodenum; the stomach seems to be a site for early hydrolysis and retention of the compound (Lu et al., 2010). These authors concluded that the upper intestine, particularly the jejunum, play the major role in flavonoid absorption (Lu et al., 2010). However, other studies have shown that for apigenin, amounts absorbed were the highest in the colon (40%) and the lowest in the terminal ileum (21%) (Chen et al., 2003). *In vivo* intestinal absorption was mediated by both passive and active carrier-mediated transport in the duodenum and jejunum, as well as concentration-dependent membrane permeability (Zhang et al., 2012). Absorption in the ileum and colon was largely *via* passive transport, in a concentration-independent manner (Zhang et al., 2012).

Some flavonoids, including apigenin, are able to modulate intestinal smooth muscle peristalsis–potentially facilitating their absorption(Gharzouli and Holzer, 2004). *In vitro* studies in guinea pigs show that apigenin interferes with muscle excitation and/or excitation-contraction, to decrease distension sensitivity and peristaltic performance in a concentration-dependent manner, thereby advantaging the absorption process (Gharzouli and Holzer, 2004). Biliary excretion and recycling enables a significantly increased half-life of the compound and prolonged exposure of the intestinal mucosa (Chen et al., 2003; Min et al., 2017). In addition to enterohepatic recycling, apigenin can also undergo local enteric recycling, in a concentration and metabolite-dependent manner (Hu et al., 2003). These enterohepatic/enteric recycling processes will tend to favor the persistence of apigenin in the gastrointestinal tract but reduce net systemic absorption.

### Variability in Apigenin Absorption, and Bioavailability

Intra-individual variability of small molecule absorption in the intestinal tract is in part related to the presence and activity of efflux pumps within the intestinal epithelium, such as the ABC transporters. ABC transporters limit flavonoid bioavailability by mediating efflux from intestinal cells into the lumen for excretion (Ravisankar et al., 2019). In Caco-2 cell monolayer models, potential dietary concentrations of apigenin (0.01–1.0μmol/L) significantly inhibited the expression of the ABC transporters ABCB–MDR1 (P-glycoprotein), ABCC–MRP2, ABCC-MRP3, and ABCG–BCRP (Ravisankar et al., 2019). This effect was synergistic with co-treatment of quercetin–another plant flavonoid (Ravisankar et al., 2019). This inhibition is believed to be mediated by directly blocking the ATP-binding site, or allosteric mechanisms (Ravisankar et al., 2019). Apigenin and quercetin enhance their own and each other’s bioavailability by downregulating the activity of ABC transporters (Ravisankar et al., 2019). Of interest to the tissue distribution patterns of apigenin, to be discussed below, *in vitro* studies of this small molecule indicate it is also able to bind and inhibit the MRP efflux protein on human erythrocytes (Łania-Pietrzak et al., 2005).

Apigenin-specific pharmacokinetic parameters from four comprehensive studies using intact animal models are presented in Table 1. Relative bioavailability (F) of oral apigenin can be calculated by dividing the area under the curve (AUC) of an orally administered subset, over that of an intravenously administered subset. Using published values for the oral AUC (Teng et al., 2012) and IV AUC (Wan et al., 2007) and correcting for different doses, this represents ∼30% bioavailability (976.29ng*h/ml/3313.10ng*h/ml). Oral bioavailabilities of small molecules in humans below 50% are considered to be low (Kim et al., 2014). After oral dosing the maximal circulating concentration (C_max_) was reached after a time (T_max_) of 0.5–2.5h, with considerable variation in the C_max_ reported in different studies. The highest C_max_ reported was 1330 ± 0.24ng/ml with an oral dose of 60mg/kg (Ding et al., 2014). Following IV administration of apigenin (20mg/kg) in rats, a mean residence time (MRT) of 0.65 ± 0.5h was calculated (Wan et al., 2007). Values for the circulating half-life (T^1^/_2_) for apigenin in these studies ranged from 1.8–4.2h with an average value of 2.52 ± 0.56h (Wan et al., 2007; Chen et al., 2011; Teng et al., 2012; Ding et al., 2014). With systematic dosing this would translate to reaching steady state within ∼12h.

**TABLE 1:** Pharmacokinetic parameters of apigenin in the literature. Four kinetic studies on oral and IV administered apigenin in rat models have been published. Doses ranged from 13.5 to 60 mg/kg. Significant variability was observed between peak plasma concentrations and the respective T_max_. Relative oral bioavailability (F) of apigenin is low, at ∼30%. Note: parameters were calculated to equivalent units.

Study data source	Model	Admin	Molecule	Dose (mg/kg)	C_max_ (ng/ml)	T_max_ (h)	AUC(0-t) (ng*h/ml)	T^1^/_2_
Teng et al. (2012)	Rats	Oral	Apigenin	13.5	42 ± 2	0.50±0.01	659 ± 25	2.11 ± 0.03
			Glucuronide metabolite		43 ± 4	1.23 ± 0.13	351 ± 13	4.69 ± 0.05

			Sulfonate metabolite		13 ± 2	1.07±0.09	20 ± 2	10.97 ± 0.13

Ding et al. (2014)	Rats	Oral	Apigenin	60	1330 ± 240	2.5±0.33	11763 ± 1520	4.198 ± 0.29
Wan et al. (2007)	Rats	IV	Apigenin	20	10934 ± 1730	IV admin (N/A)	3312 ± 473	1.75 ± 1.18
			Luteolin metabolite		78.16 ± 26.23	0.08	28.73 ± 11.33	0.97 ± 1.25
Chen et al. (2011)	Rats	IV	Apigenin-7-O-glucoside	18	0.68 ± 0.04	IV admin (N/A)	1.34 ± 0.11	2.03 ± 1.32

### Tissue Distribution of Apigenin After Systemic Uptake

In terms of plasma protein binding affinities, fluorescent spectroscopic quenching and molecular modeling analyses suggest apigenin binds to human serum transferrin glycoprotein at the Fe^3+^-binding site (Zhang et al., 2013). Specifically, this interaction is mediated by electrostatic and hydrogen bonding between the C5 and C7 hydroxyl groups of apigenin’s B-ring, and Lys-291 and Tyr-188 of transferrin (Zhang et al., 2013). There is additional hydrogen-bonding between the carbonyl of apigenin’s C-ring, and transferrin’s Arg-124 (Zhang et al., 2013).

Structural, physiological, and clinical data suggest that apigenin distributes to tissues well–as would be expected due to its high molecular lipophilicity and plasma binding capacity. Apigenin has a large Volume of Distribution (Vd) *in vivo*. Following IV administration of 20mg/kg of apigenin in rats, the Vd of apigenin was 15.75 ± 11.73L/kg–much larger than the Total Body Water (TBW) of rodents, at 0.67L/kg (Wan et al., 2007). Another study using 5.4mg/kg IV apigenin in rats showed a comparatively lower Vd at 2.07 ± 0.14L/kg, and a Vd of its metabolite luteolin at 0.868 ± 0.1L/kg–however both values are still above the TBW of rodents, indicating distribution and accumulation into tissue (Chen et al., 2012; Ninfali et al., 2013).

Lipinski’s “Rule of Five” points to whether the physical properties of chemical compounds increase their propensity of being orally active drug candidates. According to the criteria, an orally active drug cannot have a logP >5 (i.e., the drug cannot be too lipophilic). The logP of apigenin is 2.84, making it a lipophilic agent that is permeable to drugs membrane and has the ability to bypass the blood-brain barrier, but still remains orally active (Yang et al., 2014).

Specific tissues that apigenin distributes to include gastrointestinal and hepatic tissues (Lu et al., 2010). Following oral administration of *Chrysanthemum* plant extracts in rats, apigenin and luteolin were observed to distribute to tissues throughout the gastrointestinal tract–from the stomach to terminal colon - following systemic absorption and circulation (Lu et al., 2010). Both luteolin and apigenin concentrations were 3–10X higher in the jejunum, as compared to other segments of the intestines (68.2nmol/g and 84nmol/g for the two flavonoids, respectively), (Lu et al., 2010). Extracts of oral *Eclipta prostrata* in rats indicate that apigenin and luteolin accumulate most in the liver, 10 min following oral administration (Du et al., 2018). The formation rates of glucuronated and sulfonated apigenin in the liver occurs at a faster rate than apigenin excretion, by 2.5–6X and 4–6X for the respective metabolites–directly contributing to the flavonoids’ ability to accumulate in tissue(Tang et al., 2017).

### The Cellular Pharmacodynamics of Apigenin With Neoplasias as the Target

As a small molecule (MW 270g/mol), it is not surprising that apigenin interacts with multiple cellular components. However, there is accumulating evidence that these varied interactions allow apigenin to modulate cellular regulatory pathways in ways that alter the behavior of both normal and neoplastic cells, particularly those of epithelial origin. This may depend on the state of lineage differentiation; we have shown that the ability of apigenin to upregulate the cell-surface regulator CD26/DPP4 on colon epithelial cells depends on the degree of differentiation in a series of carcinoma cell lines (Lefort et al., 2020).

**FIGURE 3:**
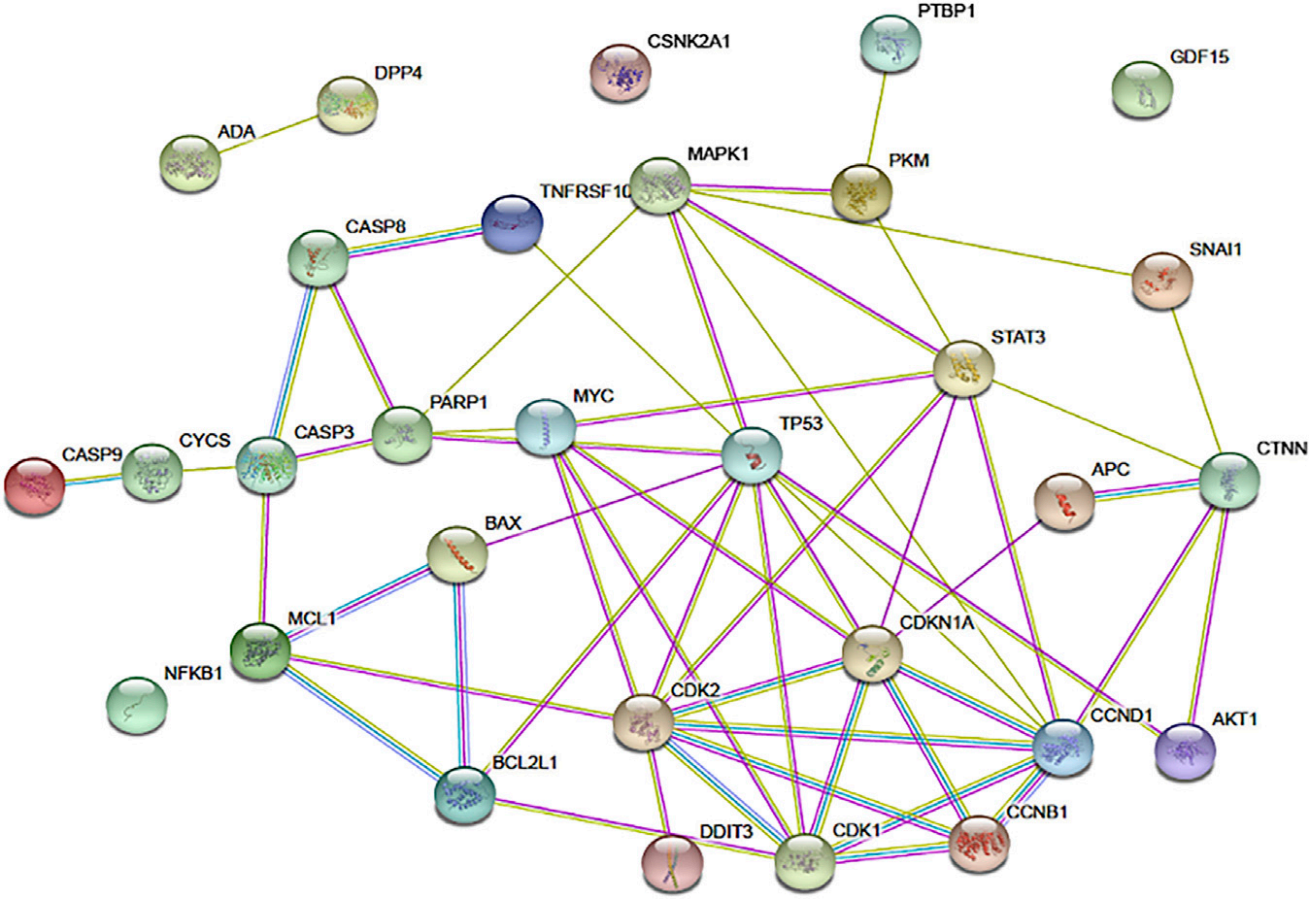
The network of connections between regulatory pathways and molecules that are known to be impacted by apigenin. The functional protein association tool STRING was used to map the inter-connectedness of impacted proteins, identified through physical protein interactions in *Homo sapiens*, as shown. Connections between nodes are color coded depending on the interaction type: blue, curated databases; fuchsia, experimentally determined; green, textmining; light purple, protein homology. Connections within this network for DPP4, ADA, CSNK2A1, and CDF15 are not shown, but published data on non-physical interactions (e.g., gene co-expression) indicate that such links exist.

**FIGURE 4:**
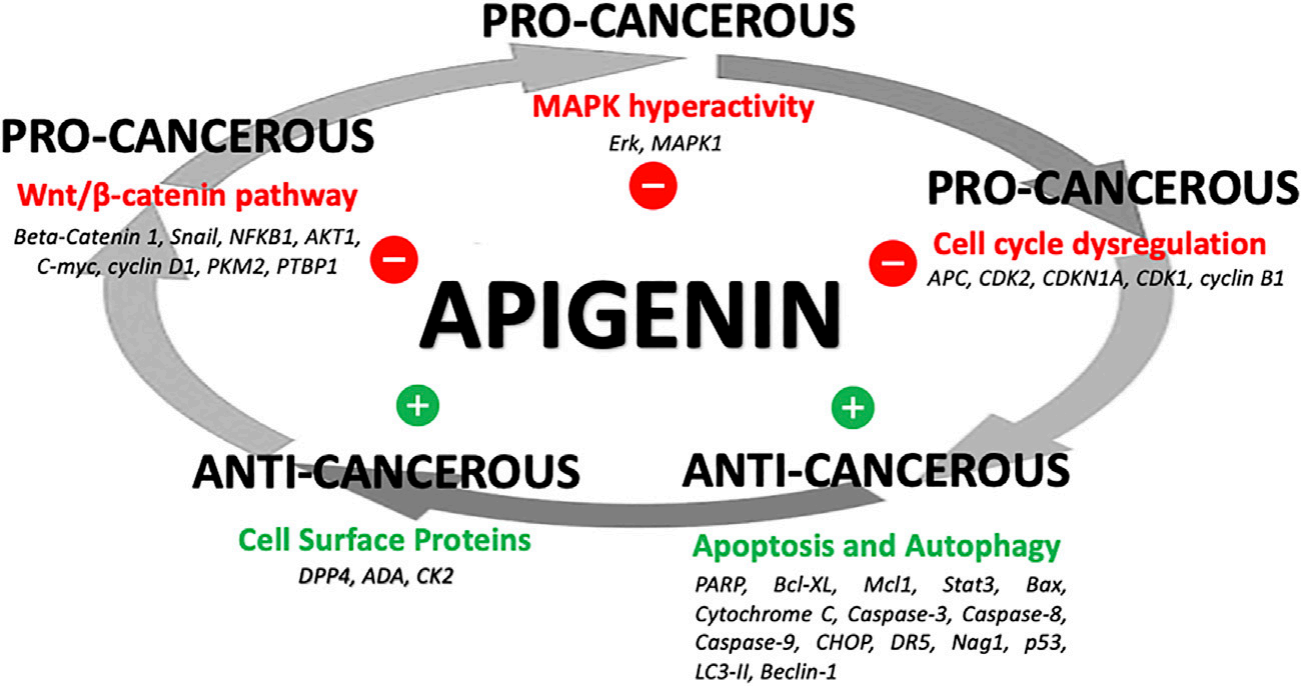
Apigenin influences on cancer processes. A summary of how apigenin is a key regulator in a number of linked cellular pathways, inhibiting pro-cancerous activity or promoting anti-cancerous activity, yielding a potential overall anti-cancer effect.

The network of cellular regulatory proteins known to be impacted by apigenin is shown in Figure 3. This includes a number of pathways relating to the cell cycle, growth, proliferation, autophagy, and apoptosis–many of which are intrinsically linked. Indeed, apigenin has a plethora of actions on intracellular pathways that underlie its potential for a net anti-cancer effect (Figure 4). Some of the affected proteins are at the cell surface, such as the dipeptidyl peptidase DPP4 (also referred to as CD26) and its bound ligand ecto-adenosine deaminase (ADA), which we have examined extensively for their roles in tumor expansion and metastasis (Tan et al., 2006; Lefort and Blay, 2011; Cutler et al., 2015). The peptidase activity of DPP4 modulates the bioactivity of the chemokine CXCL12, which acts upon the cell-surface receptor CXCR4 to control both the propensity of colorectal and other carcinoma cells to accumulate in their secondary tissue sites and form metastasis (Richard et al., 2006; Richard and Blay, 2008). This may be one route by which apigenin or related ligands could have an effect in constraining metastasis (Lefort and Blay, 2011; Lefort and Blay, 2013). However, the ability of apigenin to regulate the function of cell-surface proteins is not directly exerted at the external face of the plasma membrane. For example, the ability of apigenin to upregulate DPP4 depends on inhibition of an intracellular kinase, casein kinase 2 (CK2) (Lefort et al., 2020).

Indeed, apigenin primarily acts on intracellular protein targets such as CK2. Firstly, apigenin impacts proximal signaling through MAPK protein kinases. Hyperactivity of the MAPK pathway is linked to cancer proliferation and progression (Dhillon et al., 2007). HCT116 colon cancer cells treated with apigenin show increased MAPK phosphorylation (Dross et al., 2003). Apigenin induced 15 and 4.3-fold increases in expression of phosphorylated ERK and p38, respectively (Dross et al., 2003). This led to significantly increased transcriptional activation of downstream effectors in the MAPK pathway in a dose-dependent manner–including a 7.4-fold increase in the phosphorylation of Elk (a phosphorylation target of ERK) and a 3.2-fold increase in the phosphorylation of activating transcription factor-2 (ATF-2, a phosphorylation target of p38) (Dross et al., 2003). These data suggest apigenin’s cellular regulatory activity is in part mediated by modulating phosphorylation events of the MAPK pathway (Dross et al., 2003).

Apigenin can also modulate different aspects of the Wnt/β-catenin signaling pathway. This pathway regulates cellular behaviors including migration and the formation of tissue structures, and plays a key role in both the formation and progression of cancers, including colorectal carcinoma (Yuan et al., 2020; Bugter et al., 2021; Caspi et al., 2021). The transcription factor Snail promotes Wnt target gene expression, and downstream interactions with beta-catenin–ultimately promoting growth and proliferation (Stemmer et al., 2008). Treatment of HCT116 cells with 10–20μmol/L of apigenin led to decreased expression of Snail (also a regulator of the epithelial-to-mesenchymal transition of cancer cells) and NF-κB (a transcriptional regulator of Snail) at the RNA and protein level in a dose-dependent manner (Tong et al., 2019). In SW480 cells, apigenin inhibited *β*-catenin signaling activation in a dose-dependent manner - blocking *β*-catenin nuclear entry, expression of the Wnt proto-oncogene and downstream effector genes (e.g., C-myc, cyclin d1) (Xu et al., 2016). In HCT116 cells, apigenin affected cancer cell metabolism in a multi-modal manner *via* the Wnt/β-catenin pathway. Apigenin can directly bind and allosterically inhibit tumor-specific pyruvate kinase M2 (PKM2)–blocking glycolysis (Shan et al., 2017). However it can also inhibit expression of PKM2 at the RNA and protein level by blocking the β-catenin/c-Myc/PTBP1 pathway (Shan et al., 2017).

Apigenin interferes with cellular proliferation by effects on intracellular protein targets. An effect of apigenin on the cell cycle was observed in three different human colon cancer cell lines: SW480, HT-29 and Caco-2 (Wang et al., 2000). Cells treated with apigenin were found to be stalled at G2/M phase in the cell cycle–a phenomenon that was both time and dose-dependent (Wang et al., 2000). As the number of cells stalled at G2/M increased with time and apigenin concentration, the percentage of cells in G1 phase decreased (Wang et al., 2000; Ai et al., 2017; Wang and Zhao, 2017). This translated to 64% of SW480 cells, 42% of HT-29 cells and 26% of Caco-2 cells in G2/M arrest. Greater levels of cell cycle arrest correlated with lower IC50 concentrations of apigenin (*i.e.,* SW480 cells had the smallest IC50) (Wang et al., 2000). Apigenin treatment resulted in a significant reduction of the kinase activity of p34 (a key cyclin-dependent kinase during G2/M), p34 protein expression, and cyclin B1 protein expression (Wang et al., 2000). Apigenin may also mediate G2/M stalling by upregulation of the CDK1 inhibitor p21, and subsequently downregulating CDK1 (Takagaki et al., 2005; Oishi et al., 2013). Alternatively, *in vitro* proteomic binding assays in HT-29 cells suggest apigenin directly binds ribosomal protein S9 (RPS9), and that knockdown of RPS9 induces G2/M arrest by downregulating and destabilizing CDK1 (Oishi et al., 2013).

Finally, through its intracellular actions apigenin has effects on cellular survival by altering apoptosis and autophagy pathways. Apigenin-mediated apoptosis involves diverse regulators of cell death. Apigenin suppresses cell proliferation and induces apoptosis in a caspase- and concentration-dependent manner in colorectal HT29, COLO320, DLD-1, and HCT116 cells (Maeda et al., 2018). Notable effects include the following: i) marked increases in the cleavage of PARP, ii) downregulation of anti-apoptotic Bcl2 proteins (Bcl-xL and Mcl-l) *via* apigenin-directed inhibition of STAT3 (a Bcl2-activating kinase), iii) increased expression of pro-apoptosis regulators of both the intrinsic (Bax proteins, Cytochrome C, caspases -3,-8, -9) and extrinsic (CHOP, DR5, caspase-8) pathways, iv) increased expression of pro-apoptotic proteins NAG1 and p53, and v) increased expression of the cell cycle inhibitor p21 (Horinaka et al., 2006; Zhang et al., 2009; Zhong et al., 2010; Ai et al., 2017; Wang and Zhao, 2017; Maeda et al., 2018).

In p53-mutant HT29 human colon adenocarcinoma cells, 90μmol/L of apigenin over 72h led to apoptosis in 24.92% of cells - as well as 2.6-fold and 3.7-fold increases in caspase-3 and caspase-8 mRNA expression, respectively, (Zhang et al., 2009). The effects of apigenin on autophagy pathways in colorectal cancer cells is less clear. However, the presence of autophagosomes and the autophagy marker LC3-II were observed upon apigenin treatment in HCT116 human colon cancer cells (Zhang et al., 2009). Apigenin’s effects on apoptosis and autophagy may be mediated by destabilizing a common pathway involving PI3K/Akt/mTOR–which is known to promote tumor cell survival (Chen et al., 2019).

### Metabolism and Recycling of Apigenin and its Metabolites

In addition to its poor solubility, apigenin is also poorly bioavailable in part due to extensive first-pass metabolism and phase II conjugation by hepatic UDP-glucuronosyltransferase (UGT; specifically UGT1A1 and potentially UGT2B1) and sulfotransferase (SULT) enzymes (Wang et al., 2009). Following intestinal metabolism, the aglycone form of apigenin is more rapidly absorbed when further metabolized into phase II metabolites *via* glucuronidation and sulfation–also an essential step for the enterohepatic recycling of this flavone (Liu et al., 2003; Srinivas, 2015). In rat microsomal models, apigenin undergoes phase II metabolism to produce three different monoglucuronidated species, and a monosulfonated species (Gradolatto et al., 2004). Metabolism is 5.5-fold faster in the jejunum as compared to the colon, and both intestinal and liver microsomes are saturated at an apigenin concentration of 100μmol/L (Chen et al., 2003) *In vitro* assays on human liver microsomes suggest apigenin glucuronidation occurs at a much faster rate than sulfonation, and this may translate to different *in vivo* rates of phase II metabolism (Cai et al., 2007).

Further, metabolism to glucuronide and sulfonide species occurs to a greater extent in intestinal tissue, as compared to hepatic tissue (Cai et al., 2007). Apigenin also undergoes phase I metabolism *via* CYP1A2, and to a lesser extent CYP3A4 and CYP2B–generating the 3′-hydroxylated product luteolin (Breinholt et al., 2002). *In vitro* models show induction of CYP1A and CYP2B led to a 39% increase in luteolin formation, and luteolin formation was strongly inhibited by blocking the metabolic activity of CYP2E1, CYP3A, CYP2B, and CYP2C9 (Gradolatto et al., 2004). Luteolin phase II conjugates in rats include four monoglucoronidated species, and two monosulfoconjugates (Gradolatto et al., 2004).

Table 2 outlines the major CYPs affecting apigenin metabolism, as well as common compounds and/or medications known to be inducers or inhibitors of these phase I hepatic enzymes (Kleinschmidt and Delaney, 2015). Patients taking these medications would encounter elevated (with CYP inhibitors) or reduced (with CYP inducers) plasma levels of apigenin if taken concomitantly.

**TABLE 2:** Apigenin as a substrate for phase I CYP enzymes. Based on studies looking at the induction or inhibition of luteolin production (a primary metabolite of free apigenin), data suggests that apigenin metabolism is induced by CYP1A and CYP2B enzymes, and inhibited by CYP2E1, CYP3A, CYP2B, CYP2C9 (Breinholt et al., 2002; Gradolatto et al., 2004). If these effects are corroborated *in vivo*, drug interactions involving medications that induce or inhibit these CYP enzymes would lead to increased apigenin metabolism (and lower plasma levels), or decreased metabolism (and increased plasma levels), respectively.

Apigenin metabolizer	Common inducers	Common inhibitors
CYP1A2	*Nafcillin, Rifampin, Primaquine, Carbamazepine, Secobarbital, Pentobarbital, Phenobarbital, Phenytoin, Nelfinavir, Ritonavir* ^*^ *, Lansoprazole, Omeprazole, Antipyrine, Coffee, Cruciferous vegetables, Insulin, Sulfinpyrazone, St. John’s Wort, Teriflunomide*	Ciprofloxacin^*^, enoxacin^*^, Norfloxacin, Macrolides, Duloxetine, Fluvoxamine^*^, Acyclovir, efavirenz, Peginterferon-α-2a, *Amiodarone, Mexiletine, Propafenone, Ticlopidine, Verapamil, Rofecoxib, Tolfenamic acid, Allopurinol, Cimetidine, Disulfiram, Famotidine, Grapefruit juice, Methoxsalen, Oral contraceptives, Zafirlukast* ^*^
CYP2B6	*Artemisinin antimalarials, Rifampin, Carbamazepine* ^*^ *, Phenobarbital, Phenytoin, Antiretrovirals, Efavirenz, Nelfinavir, Nevirapine, Ritonavir, Cyclophosphamide, Metamizole, St. John’s Wort, Statins, Vitamin D*	*17-α-ethynylestradiol, Clopidogrel, Clotrimazole, Mifepristone (RU-486), Phencyclidine, Sertraline, Tenofovir, ThioTEPA, Ticlopidine, Voriconazole*
CYP2C9	*Rifampin, Rifapentine, Amobarbital, Carbamazepine, Pentobarbital, Phenobarbital, Phenytoin, Secobarbital, Nelfinavir, Nevirapine, Ritonavir* ^*^ *, Dexamethasone, Prednisone, Aminoglutethimide, Antipyrine, Aprepitant, Avasimibe, Bosentan, Smoking, Cyclophosphamide, Enzalutamide, Glutethimide, Nifedipine, St. John’s Wort, Statins*	*Clarithromycin, Erythromycin, Troleandomycin, Isoniazid* ^*^ *, Metronidazole* ^*^ *, Rifampin, Sulfamethoxazole, Trimethoprim, Fluoxetine, Fluvoxamine, Paroxetine, Sertraline, Felbamate, Valproic acid, Fluconazole* ^*^ *, Itraconazole, Ketoconazole, Miconazole, Sulfaphenazole, Voriconazole, Efavirenz, Ritonavir* ^*^ *, Fluvastatin, Lovastatin, Amiodarone, Cimetidine, Disulfiram, Grapefruit juice*
CYP2C19	*Analgesics, Antipyrine, Salicylates, Antibiotics, Artemisinin antimalarials, Rifampin* ^*^ *, Rifapentin* ^*^ *, Carbamazepine, Phenobarbital, Phenytoin, Efavirenz, Nelfinavir, Ritonavir* ^*^ *, Dexamethasone, Prednisone, Enzalutamide, St. John’s Wort*	*Clarithromycin, Erythromycin, Troleandomycin, Chloramphenicol, Isoniazid, Citalopram, Fluoxetine* ^*^ *, Fluvoxamine* ^*^ *, Moclobemide* ^*^ *, Paroxetine, Sertraline, Antiepileptics, Felbamate, Oxcarbazepine, Topiramate, Valproic acid, Fluconazole* ^*^ *, Ketoconazole, Voriconazole, Esomeprazole, Lansoprazole, Omeprazole* ^*^ *, Pantoprazole, Rabeprazole, Cimetidine, Clopidogrel, Grapefruit juice, Indomethacin, Oral contraceptives, Ritonavir* ^*^ *, Ticlopidine* ^*^
CYP2E1	*Acetone, ATRA, Ethanol, Isoniazid, Smoking, St. John’s wort, Styrene, Toluene*	*Clomethiazole, Diethyldithiocarbamate, Disulfiram, 4-Methylpyrazole, Orphenadrine*
CYP3A4	*Nafcillin, Rifabutin, Rifampin* ^*^ *, Rifapentine, Troleandomycin, Amobarbital, Carbamazepine* ^*^ *, Felbamate, Oxcarbazepine, Pentobarbital, Phenobarbital, Phetharbital, Phenytoin* ^*^ *, Rufinamide, Topiramate, Valproic acid, Amprenavir, Efavirenz, Etravirine, Nelfinavir, Nevirapine, Ritonavir* ^*^ *, Tipranavir, Bexarotene, Enzalutamide* ^*^ *, Imatinib, Mitotane* ^*^ *, Vinblastine, Dexamethasone, Methylprednisolone, Prednisolone, Prednisone, Alitretinoin, Antipyrine, Aprepitant, Armodafinil, Artemisinin antimalarials, Avasimibe, Bosentan, Ethanol, Gingko biloba, Metamizole, Miconazole, Modafinil, Organochlorine compounds, Phenylbutazone, Pioglitazone, St. John’s Wort* ^*^ *, Statins, Sulfinpyrazone, Troglitazone*	*Clarithromycin* ^*^ *, Erythromycin, Telithromycin* ^*^ *, Troleandomycin* ^*^ *, Azithromycin, Chloramphenicol, Ciprofloxacin, Isoniazid, Norfloxacin, Fluoxetine, Fluvoxamine, Nefazodone* ^*^ *, Norfluoxetine, Paroxetine, Sertraline, Amiodarone, Dronedarone, Quinine, Clotrimazole, Fluconazole Itraconazole* ^*^ *, Ketoconazole* ^*^ *, Miconazole, Posaconazole* ^*^ *, Voriconazole* ^*^ *, Cimetidine, Ranitidine, Amprenavir, Atazanavir* ^*^ *, Cobicistat* ^*^ *, Danoprevir* ^*^ *, Elvitegravir* ^*^ *, Fosamprenavir, Indinavir* ^*^ *, Lopinavir* ^*^ *, Nelfinavir* ^*^ *, Paritaprevir* ^*^ *, Ritonavir* ^*^ *, Saquinavir* ^*^ *, Tipranavir* ^*^ *, Amlodipine, Diltiazem* ^*^ *, Nicardipine, Nifedipine, Verapamil, Ergotamines, Ticagrelor, crizotinib, Idelalisib* ^*^ *, Imatinib, Irinotecan, Boceprevir* ^*^ *, Telaprevir* ^*^ *, Cyclosporine, Tacrolimus, Aprepitant, Chlorzoxazone, Cilostazol, cocaine, Conivaptan* ^*^ *, Ethinylestradiol, Felbamate, Fosaprepitant, Grapefruit Juice* ^*^ *, Istradefylline, Ivacaftor, Lomitapide, Mifepristone (RU-486), Nicotine, Olanzapine, Propofol, Ranolazine, Tofisopam, Zileuton*

* indicates the medication is a potent inhibitor/inducer.

Apigenin phase II conjugates are excreted throughout the gastrointestinal tract, but largely in the jejunum (Chen et al., 2003). In rat models, approximately 33% of total apigenin is excreted into the gut, while 7% is excreted into the bile (Chen et al., 2003). Assays of steady-state concentration in bile suggest significantly higher concentrations than those used initially for perfusion–indicating that apigenin has the ability to concentrate in bile (Chen et al., 2003). Similar to many plant flavonoids, apigenin is able to undergo enterohepatic recycling (Chen et al., 2003). During enterohepatic recycling, the glucuronide and sulfonate metabolites are excreted into the intestines *via* the bile or enterocytes. The metabolites can then re-convert into aglycones *via* glucuronidases or sulfatases in the colon (Min et al., 2017). In Caco-2 cell monolayer models, low concentrations of apigenin resulted in sulfonated metabolites being excreted apically to a concentrated level through high-affinity, low-capacity transport(Hu et al., 2003). Higher concentrations of apigenin resulted in more glucoronidated metabolites being excreted basolaterally (Hu et al., 2003).

### Clearance and Elimination of Apigenin and its Metabolites

Apigenin’s high Vd and enterohepatic/enteric recycling processes indicate the elimination patterns of this lipophilic molecule will be delayed. Indeed, the elimination patterns in rat models using a single dose of 10mg of radiolabeled apigenin supports this (Gradolatto et al., 2005). Following oral administration, ∼50% of apigenin was recovered in urine and ∼12% in feces (Gradolatto et al., 2005). Unchanged, glucuronidated, and to a lesser extent sulfonated species, were recovered in all excrements (Gradolatto et al., 2005). Although most products were excreted within the first 24 h, about 25% of the original apigenin dose was retained 10 days after treatment (Gradolatto et al., 2005). Plasma clearance was estimated at 2ml/h (Gradolatto et al., 2005). For humans, monoglucoronidated and monosulfonated species of apigenin have been recovered from urine and identified by HPLC (Nielsen and Dragsted, 1998).

There are differences in rates of clearance between apigenin and its primary metabolite luteolin. Following 5.4mg/kg IV administration of apigenin in rats, the clearance of luteolin was 0.450 ± 0.03L/h/kg (Chen et al., 2012; Ninfali et al., 2013). Apigenin clearance was 0.065 ± 0.005L/h/kg (Chen et al., 2012; Ninfali et al., 2013). Studies using primary rat hepatocytes suggest this faster rate of elimination for luteolin is likely due to rapid metabolism by phase I metabolism, in addition to phase II metabolism by UGTs, SULTs, and COMT–whereas apigenin is mostly metabolized *via* UGT and SULTs (Chen et al., 2012; Ninfali et al., 2013).

### The Capacity for Drug Interactions Between Apigenin and Medications

As mentioned, apigenin in its aglycone form can undergo phase I metabolism by CYP enzymes. The flavonoid chemical structure (i.e., C2-C3 double bonds and A-ring hydroxylation) makes these small molecules as a class potent inhibitors of CYP1A1, CYP1A2 and CYP1B1 in yeast, rat and human models (Androutsopoulos et al., 2010; Kimura et al., 2010). Apigenin is also a potent inhibitor of CYP1A2 and CYP2C9 (Kale et al., 2008; Si et al., 2009). Concomitant use of apigenin and certain drugs (Table 2) could result in either a Class C-D drug-drug interactions, or complete contraindications as defined in the healthcare professional resource Lexicomp (Lexicomp Inc, 2021).

Potentially more worrisome are drug interactions involving medications that are substrates of CYP enzymes affected by apigenin–resulting in toxicity or supratherapeutic effects of the prescribed medications at high apigenin levels. In the context of cancer, the principal chemotherapies and/or supportive care drugs metabolized by the key CYP enzymes that are inhibited by apigenin are listed in Table 3 (Kleinschmidt and Delaney, 2015). It is important to note that where there are prodrugs that are metabolized by apigenin-inhibitable CYPs, there is a risk of subtherapeutic effects of the active drug. Similarly, drugs metabolized for elimination by CYPs inhibited by apigenin will exhibit enhanced therapeutic effects but also a higher risk of toxicity.

**TABLE 3:** CYP enzymes inhibited by apigenin, and their respective substrates of relevance to cancer treatment. Studies have indicated that apigenin is an inhibitor of CYP1A2, CYP2C9, and CYP3A4 (Kleinschmidt and Delaney, 2015). This implicates apigenin in potential drug interactions with a number of chemotherapies and medications used in the supportive care of cancer. If this flavone were to be taken in conjunction with any one of the interacting substrates, this would lead to decreased metabolism and potentially supratherapeutic or toxic plasma levels.

Enzyme inhibited by apigenin	Substrates
CYP1A2	- Nausea and vomiting
	*Alosetron* ^*^ *, Ondansetron, Ramosetron* ^*^ *, Olanzapine*
	- Cancer-induced VTE
	*Warfarin*
	- Hormones
	*Estradiol*
CYP2C9	- Chemotherapy and other cancer treatments
	*Cyclophosphamide, Tamoxifen*
	- Chemo-induced nausea and vomiting
	*Tetrahydrocannabinol*
	- Cancer-induced VTE
	*Warfarin* ^*^
CYP3A4	- Chemotherapy and other cancer treatments
	*Cyclophosphamide, Dasatinib, Docetaxel, Erlotinib, Etoposide, Gefitinib, Ibrutinibb, Ifosfamide, Imatinib, Irinotecan, Teniposide, Vincristine* ^*^ *, Tamoxifen, Flutamide*
	- Chemo-induced nausea and vomiting
	*Granisetron, Ondansetron, Haloperidol, Dexamethasone, Alprazolam, clonazepam, Diazepam, Midazolam* ^*^ *, Triazolam* ^*^ *, Tetrahydrocannabinol*
	- Cancer-induced VTE
	*Warfarin*
	- Chemo-induced Diarrhea
	*Loperamide*
	- Chemo-induced Immune-mediated reactions
	*Chlorpheniramine, Desloratadine, Ebastine* ^*^ *, Loratadine, Terfenadine* ^*^ *, Cortisol, Hydrocortisone, Methylprednisolone, Prednisone*
	- Hormones
	*Estradiol, Progesterone, Testosterone*

* Indicates the compound has a narrow therapeutic range and/or highly metabolized by the CYP enzyme

We are currently unaware of the existence of clear guidelines for potential apigenin interactions with other medications (or indeed sound evidence-based justifications for its clinical efficacy in disease situations). However, the Therapeutic Research Center Natural Medicines database for healthcare professionals has detailed professional monographs for various natural products, including parsley, German chamomile and chrysanthemum–key plant sources with some of the highest contents of apigenin found in nature (Natural Medicines TRC, 2021a; Natural Medicines TRC, 2021b; Natural Medicines TRC, 2021c).

### The Required Local Concentration of Apigenin to Exert Cellular Actions

The concentrations of apigenin required to produce a cellular effect vary substantially between different studies and the cellular response being measured, but the lowest effective concentration is within a significant proportion of the studies is in the range 1–5μmol/L (Table 4). It is notable that in this limited sample of studies, the lower threshold concentrations are seen with a 48h exposure time, whereas higher concentrations are required for treatments with a shorter (24h) exposure to the single apigenin doses used. This would be consistent with a model in which apigenin’s effects on cells involve multiple successive pathways that interact to give the final change in overall cell phenotype.

**TABLE 4:** Concentrations of apigenin necessary to produce cellular responses in human cancer cells. A representative series of examples were taken from the literature cited in this review. The main experimental readouts are indicated, although studies included more detail. All targets are human cells, and in all cases involve a single dose of apigenin except for one example in which colony formation was evaluated over seven days and apigenin was added on a daily basis (Shan et al., 2017).

Study data source	Model	Readout of interest	Typical exposure time	Lowest effective concentration	EC50 or IC50
Wang et al. (2000)	Colorectal cancer cells	Cell cycle G2/M arrest	24–48h	30μmol/L	40–70μmol/L^a^
Horinaka et al. (2006)	Multiple cancer cell lines	Cell-surface DR5 (TRAILR2)	24h	20μmol/L^b^	−
Lefort and Blay (2011)	Colorectal cancer cells	Cell-surface CD26/DPP4	48h	1μmol/L	3–30μmol/L^a^
Oishi et al. (2013)	Prostate cancer cells	Mitochondrial ANT2	24h	20μmol/L^b^	−
Xu et al. (2016)	Colorectal cancer cells	Viability, proliferation, migration	48h	5μmol/L	18–24μmol/L^a^
Shan et al. (2017)	Colorectal cancer cells	Colony formation, survival	24h, 7d	10μmol/L	28–90μmol/L^a^
Wang and Zhao (2017)	Colorectal cancer cells	Cell growth, apoptosis	24–72h	40μmol/L	78–98μmol/L^a^
Maeda et al. (2018)	Colorectal cancer cells	Proliferation, apoptosis	48h	5μmol/L	∼15μmol/L^b^
Tong et al. (2019)	Colorectal cancer cells	Viability, EMT, migration	24h	10μmol/L	34–47μmol/L^a^
Lefort et al. (2020)	Colorectal cancer cells	Cell-surface CD26/DPP4	48h	3μmol/L	∼10μmol/L^b^

a Depending on parameter measured.

b Estimated from data provided.

We will use 1–5μmol/L as the minimal local extracellular apigenin concentration that has to be attained at a particular location *in vivo* in order to provoke a cellular response in the cell population of interest, and the necessary plasma concentration. This is likely to be a generous estimate as most experimental studies (and all listed in Table 4) use only a single treatment, which would result in a progressively declining apigenin concentration. However, appropriate *in vivo* dosing is repetitive and offsets any degradation or metabolism of the agent. In our work on down-regulation of DPPIV on human colorectal cancer cells by adenosine, it was necessary to use a single dose concentration of 300μmol/L to produce a robust effect. However, allowing for degradation half-life and using lower doses (and a reduced overall amount) successively over the 48h response period reduced the exposure level, with no change in effect, to 12.5μmol/L or lower, a 24-fold alteration in requirement (Tan et al., 2004). There has been no comparable study of the effect of dosing frequency for apigenin, but based on a biological half-life of ∼2.5h, a spaced dosing regimen would be optimal for a response that takes ∼48h to develop and involves the sequential participation of successive cellular pathways. As well, there is no active mixing in cell culture models, leading to an unstirred fluid layer next to the cell surface which reduces the concentration of ligand that is available to interact with cells (Shibayama et al., 2015; Kono et al., 2016). This again necessitates higher concentrations in the bulk fluid medium than are truly necessary for a response. This of course is not the case in perfused tissue. For both of these reasons, we believe the 1–5μmol/L target to be a very conservative estimate for the extracellular apigenin concentration required for activity *in vivo*. The cell lines used in these preclinical studies (Table 4) are immortalized cell lines from tumor sources, so this rationale is applicable to affecting cancer cells.

### The Utility of Apigenin Taken Orally in its Natural Herbal Form

Humans consume on average between 0.45–1.17mg of apigenin daily (Meyer et al., 2006; Somerset and Johannot, 2008). In the bioavailability study of Meyer and colleagues, adult volunteers consumed a meal containing 2g parsley/kg body weight–corresponding to 149 ± 35g of parsley (mean body weight 75kg) and providing an average of 18 ± 4mg (66 ± 15μmol) of apigenin (Meyer et al., 2006). Such dosing led to mean plasma concentrations that exceeded 100nmol/L between 6–9h after parsley consumption and reached a maximum of 337nmol/L (Meyer et al., 2006) These established plasma levels (0.1–0.3μmol/L) begin to approach the target range of 1–5μmol/L. However, this corresponds to almost two cups of this low density herb, representing in the region of 15 to 40-fold the normal daily intake. Attaining an apigenin level with sufficient capacity to influence cell function at systemic locations would require of the order of 100-fold the regular daily dietary intake of even apigenin-rich foods. As a therapeutic or assistive option, this would be unreasonable - especially in cancer populations, where malnutrition secondary to nausea, vomiting, and poor appetite is common. Thus, the use of pure apigenin extracts, dry-powder capsules, and/or IV treatments are essential to take advantage of the beneficial effects of this flavone.

### The Utility of Apigenin Taken Orally as a Semi-purified Preparation

Comprehensive *in vivo* studies looking at the pharmacokinetic properties of purified apigenin given by the oral route have not yet been conducted in humans. In rats, doses of apigenin assessed were 13.5 and 60mg/kg (see Table 1 above). While it is difficult to compare the gastrointestinal physiology of rats to humans, in a 70kg adult person, this is equivalent to apigenin doses of 0.9 and 4.2 g, respectively. Compared with the dosing with dietary parsley mentioned above, this would be approximately a 50–240-fold reduction in bulk intake yet should take plasma concentrations into the bioactive range in humans (by simple extrapolation, to at least 5μmol/L). Measurements in rats at the higher oral dose gave a maximal plasma concentration of 1330ng/ml(Ding et al., 2014). With a molecular mass for apigenin of 270g/mol, this equates to 4.9μmol/L. Although an approximation based upon limited data, both the rat model and extrapolation from human data therefore suggest that doses used and approved in experimentation reach systemic levels into the range to achieve biological effects. Indeed, it should be noted that substantially higher doses of apigenin of up to 300mg/kg have been used in mouse models, with no overt toxicity or effect on body weight up to 68 days of exposure (Ai et al., 2017; Tong et al., 2019).

Taking the higher 4.2g dose and with the density of apigenin being ∼1.5g/ml (National Center for Biotechnology Information, 2021), this would correlate to a product volume of 2.8ml. The largest commonly used capsules hold 1.37ml of material(Medisca, 2021). Therefore, 2.8ml of apigenin powder (which represents the dose we predict should yield a 5μmol/L plasma concentration of apigenin) would require two large capsules of purified flavone to provide this oral dosage in humans. This is feasible.

Therefore in principle it should be possible, using oral dosing of apigenin capsules, to reach circulatory concentrations that are able to influence the biology of systemic targets. Circulatory apigenin should be available to act through the extracellular milieu for a substantial time. As indicated above there is a persistence of 6–9h after oral ingestion from human parsley consumption (Meyer et al., 2006) and a T^1^/_2_ of 2.1–4.2h in rodents (Teng et al., 2012; Ding et al., 2014). With reasonable dosing several times per day, effective levels could be maintained for long enough a period to exert effects on the cell behaviors described in this review.

The oral dosage form necessary in a therapeutic context therefore needs to be of a reasonably purified form of apigenin itself, since the doses of apigenin that are therapeutically relevant cannot be achieved by oral consumption of whole plant products such as parsley. Parsley leaf itself is available in capsule form as a health supplement, with the typical recommended ingestion amount being 2 capsules for a total dose of 900mg. Using an apigenin content for dried parsley of 45mg/g (Sung et al., 2016), this would equate to a daily dose of approximately 40mg apigenin, less than 1% of what is needed for a meaningful clinical outcome on cancer cell behavior, although it may have other beneficial effects on health. Crude plant products are therefore not adequate in this context.

Purified forms of apigenin itself (CAS# 520–36–5) are however readily available for research purposes and could be upscaled for human ingestion following appropriate protocols. Purity levels of 95–98% are available from multiple suppliers globally, both sourced from natural products (citrus, chamomile) and synthetic. Naturally sourced apigenin is purified via chromatographic methods and produced in powder or recrystallized forms. Incorporation into capsules at a typical 97% purity level would not compromise feasibility and other than the capsule casing comprised of cellulose or gelatin, no other excipients (binding agents, lubricants, fillers, colors) would be required.

### Parenteral Delivery of Apigenin

While IV administration of apigenin would be disfavoured in humans as not being overtly justified, it would be predicted to enable biologically active concentrations. Indeed, IV delivery of apigenin (20mg/kg) in rats yielded a C_max_ of 11.0 ± 1.7μg/ml (Wan et al., 2007), which represents a plasma concentration in excess of 40μmol/L, sufficient to produce a response in all of the cell systems studied (Table 4).

### Options to Enhance the Bioavailability of Apigenin Taken Orally

If taken in a [semi]-purified form for health beneficial purposes, additional measures can be used to ensure circulatory apigenin levels reach the higher concentrations apparently necessary (in single-dose *ex vivo* studies) for certain cellular responses (Table 4). Apigenin can be generated in different dosage forms beyond simple encapsulation. Improved bioavailability of oral dosage forms have been evaluated by using apigenin-loaded water-in-oil-in-water emulsions (Kim et al., 2016). These emulsions were tested using *in vitro* simulations of digestion, confirming that the carriers enable the delivery of bioactive compounds in a water phase, while minimizing degradation and potentially improving *in vivo* bioavailability (Kim et al., 2016). In another effort to improve solubility in oral dosage forms, apigenin and its potassium salt form were compared using Caco-2 cell monolayers (Sánchez-Marzo et al., 2019). While solubility was vastly improved in the salt form, both apigenin and its salt exhibited similar intestinal apparent permeabilities (Sánchez-Marzo et al., 2019).

Emerging technologies to enhance apigenin oral delivery include nanoparticles, also using surfactants for enhanced drug solubility. Such nanoparticle delivery systems increase bioavailability in a number of ways: i) protection from chemical degradation in the gastrointestinal tract, ii) improved absorption via the lymphatic system, iii) protection from first-pass metabolism, iv) sustained release, and v) site-of-action targeting (e.g., solid tumor) (Leonarduzzi et al., 2009; Wang et al., 2013). Three examples of these nanoparticle technologies include mixed micelle systems (Zhang et al., 2017), pharmacosomes (Telange et al., 2017), and self-microemulsifying drug delivery systems (Zhao et al., 2013). The improved pharmacokinetic parameters of these nanocarrier systems would translate to improved delivery of apigenin as a pharmacologic agent. There is also the potential to increase efficacy of apigenin actions. Indeed, liposomal formulations of apigenin have been shown to enhance antineoplastic efficacy against colorectal cancer both *in vitro* and *in vivo* (Banerjee et al., 2017).

Furthermore, there are methods under development that would more effectively deliver apigenin to the intended site of action following oral administration. For example, TPGS (d-alpha-tocopheryl polyethylene glycol 1,000 succinate) is a water-soluble co-polymer derived from vitamin E. This forms amphiphilic nanoparticles comprised of a hydrophilic PEG head and hydrophobic tocopherol tail, that have unique advantages as drug carriers (Gorain et al., 2018). In the context of cytotoxic chemotherapeutics, paclitaxel-loaded TPGS nanocarriers enable enhanced drug solubilization, inhibition of P-gp-mediated drug resistance (paclitaxel being a substrate of P-gp), improved cancer cell permeability, promotion of cell cycle arrest and apoptosis (Gorain et al., 2018). Mixed micelles including TPGS for the co-delivery of synergistic chemotherapeutics show promise in diverse oncopathologies. For example, polylactide-co-glycolide/D-alpha-tocopherol polyethylene glycol 1000 succinate (PLGA/TPGS) nanocarriers are able to co-deliver docetaxel and salinomycin to strategic benefit (Gao et al., 2019). These carriers were successful in delivering and maintaining 1:1M ratios of these synergistically cytotoxic agents both *in vitro* and *in vivo* to breast cancer cells and their stem cells (Gao et al., 2019). Similar positive results have been shown using TPGS mixed micelles targeted toward liver cancer (docetaxel and piperine), as well as ovarian cancer (paclitaxel and fenretinide) (Ding et al., 2020; Wang et al., 2020). Such a TPGS-based dual delivery system may prove advantageous in combining apigenin with primary chemotherapeutics.

Bioactive Self-Nanoemulsifying Drug Delivery Systems (Bio-SNEDDSs) have been used with apigenin and are effective in improving solubility and bioavailability of the flavonoid, both *in vitro* and *in vivo* (Kazi et al., 2020). Nanoemulsion of apigenin results in decreased drug particle size that can be absorbed to a greater extent and potentially bypass degradation in the gastrointestinal tract (Kazi et al., 2020). Studies in rat models have indicated that Bio-SNEDDS result in the formation of nanosized and homogenous apigenin-loaded particles that lead to significantly improved C_max_ concentrations and AUCs (by 105 and 91%, respectively) compared to administration of pure apigenin powder (Kazi et al., 2020). As well, the bioactive and nutritive lipid excipients in the delivery system may in fact have additional therapeutic effects (Kazi et al., 2020). Such a delivery approach may further increase the feasibility of using oral apigenin clinically.

While our focus here is on oral delivery of apigenin and its formulations, nanoparticles would also be advantageous if an intravenous route were being considered and would likely circumvent problems such as apigenin’s rapid crystallization into plasma post-delivery (Karim et al., 2017). Injectable nanoparticle drug delivery systems include lipid nanocapsules, polymeric nanoscapsules, and liposomes (Karim et al., 2017). These confer a number of benefits, including: i) good encapsulation of apigenin and loading capacity, ii) stability during storage, iii) reduced cytotoxicity, and iv) extended release of apigenin due to a less pronounced carrier burst effect (Karim et al., 2017).

## Discussion

### Challenges and Opportunities in the Therapeutic Use of Apigenin

Careful examination of the available data indicates that it is unlikely that dietary ingestion of apigenin-containing plant materials–even those with very high levels such as parsley–will lead to biologically meaningful effects on cells through the vascular route without heroic levels of intake. This does not completely exclude the possibility that local high concentrations of apigenin acting directly from the gastrointestinal lumen could affect intestinal tissues in the gut wall, including primary tumors. The potential for locally efficaceous concentrations could be examined by local tissue measurements of apigenin uptake, perhaps using the microdialysis approach that we introduced for measuring metabolites in solid tumors (Blay et al., 1997).

Use of apigenin supplements, with purified apigenin in capsules, can achieve biologically relevant plasma concentrations that would be capable of influencing cellular behaviors. From the therapeutic perspective the flavonoid structure, although well handled by the body as a natural product, has challenges in terms of delivery and subsequent metabolism of the bioactive molecule. The molecular characteristics of apigenin and related flavones lead to poor solubility, moderate permeability, and chemical instability (Zhao et al., 2019). These properties can be attributed to a number of structural characteristics of flavones, including i) a planar structure due to double bonds between positions 2 and 3 of the flavones, resulting in tight molecular arrangements and poor penetration, ii) frequent glucoronidation and sulfation of flavone hydroxyl groups in the gastrointestinal tract, leading to luminal efflux and inadequate net oral absorption, and iii) sensitivity of hydroxyl, ketone and unsaturated double bonds to pH and enzyme attack (Zhao et al., 2019). This constitutes a hurdle to an effective therapeutic strategy.

However, the chemical simplicity of apigenin and the presence of modifiable groups also facilitates strategies to modify and improve apigenin’s delivery and access to the target location. The interference due to basic physicochemical characteristics can be bypassed with the development of prodrugs, glycosylation, the use of absorption enhancers such as cyclopentadecalactone, and the nanotechnologies discussed above (Gorain et al., 2018; Gao et al., 2019; Zhao et al., 2019; Ding et al., 2020; Kazi et al., 2020; Wang et al., 2020). Indeed, there is an opportunity to capitalize on the unique features of this small molecule. The flavone structure is a ready scaffold for further modification using medicinal chemistry (Singh et al., 2014). In our own work, we have shown that changes in hydroxylation or glycosylation have dramatic effects on the ability of apigenin to upregulate CD26, and thus potentially affect hallmarks of cancer (Lefort and Blay, 2011; Lefort et al., 2020). Therefore, using apigenin as a lead compound in drug development may be a valuable option for optimizing a small molecule to fully capture the beneficial effects that have been identified.

### Caveats in the Therapeutic Use of Apigenin in Oncology Based Upon Current Knowledge.

Whether the administration of apigenin will indeed achieve clinically beneficial outcomes requires focused studies that combine direct measurements of plasma apigenin levels with clinical outcomes during chemotherapy. Although studies of oral and parenteral administration of apigenin in rat models have produced some valuable pharmacokinetic data (Wan et al., 2007; Chen et al., 2011; Teng et al., 2012; Ding et al., 2014) there has been very little direct pharmacokinetic assessment in humans and it has been restricted to dosing with the dietary natural product such as parsley (Meyer et al., 2006). Full assessment of apigenin and metabolites, and particular intra-patient variability given the variation in apigenin absorption and bioavailability noted above, need to be factored in alongside assessment of safety and toxicity in phase I clinical trials.

There is substantial evidence for beneficial anti-cancer effects of apigenin, particular in the context of gastrointestinal cancers (Lefort and Blay, 2013). However, much of the preclinical *in vitro* data utilizes highly selected lines of cancer cells that have been adapted to culture and that in monolayer may not well represent the behavior of cells within 3-dimensional tissue frameworks (Lowthers et al., 2003; Howes et al., 2014). These cell populations may have different genotypes and behaviors compared with primary tumor isolates. As well, few *in vivo* studies use cancers implanted orthotopically, which better recapitulate the disease evolution compared to subcutaneous tumors (Lensch et al., 2002; Flatmark et al., 2004).

In addition, most pharmacodynamic studies characterizing the mode(s) of action of apigenin involve treatment using the flavonoid as a monotherapy (Wang et al., 2000; Horinaka et al., 2006; Lefort and Blay, 2011; Oishi et al., 2013; Xu et al., 2016; Shan et al., 2017; Wang and Zhao, 2017; Maeda et al., 2018; Tong et al., 2019; Lefort et al., 2020), whereas clinical oncology uses combination regimens and the addition of a component such as apigenin would be as a further agent in addition to the combination used against the category and stage of neoplasic being addressed. However, we have found that apigenin has a valuable and potentially synergistic advantage when given together with chemotherapeutics in certain cancer readouts (Lefort and Blay, 2011), meaning that its addition to existing combinations is inherently worthy of study. As we have illustrated in this review and elsewhere (Lefort and Blay, 2011; Lefort and Blay, 2013; Lefort et al., 2020), there is a great diversity of cellular responses that has been shown for apigenin in preclinical cancer models. It is therefore important that in early studies of efficacy in humans, all relevant cellular markers of progression are evaluated. As patients may have comorbidities for which they are receiving other medications, it will also be important to take account of possible drug reactions as we have indicated in Table 3.

### Possible Beneficial Effects of Apigenin Concurrent With Chemotherapy

Although not justifiable in itself as a therapeutic strategy, the status of apigenin as an inhibitor of CYP1A2, CYP2C9 and CYP3A4 means that it may enhance rather than inhibit the action of certain cancer medications by altering their pharmacokinetics in a beneficial way. Apigenin can also improve the pharmacokinetic parameters of the chemotherapeutic agents docetaxel and paclitaxel–two microtubular depolymerization inhibitors with poor bioavailability (Kumar et al., 2015). Co-administration of 10mg/kg of apigenin to adult male rats *via* oral gavage with 40mg/kg docetaxel significantly increased docetaxel C_max_ (137%) and AUC (162%)–without affecting T^1^/_2_ (Teng et al., 2012). Co-administration of apigenin with paclitaxel led to the AUC, C_max_, MRT, and T^1^/_2_ of paclitaxel increasing with apigenin in a concentration-dependent manner, likely due to both CYP3A4 and P-gp efflux pump inhibition in the intestines (Kumar et al., 2015). These data suggest apigenin may enhance the bioavailability of taxanes–potentially requiring a lower dose of these chemotherapies for adequate clinical effect.

P-gp overexpression is a key player in multi-drug chemotherapy failure of human cancer cells. P-gp is an ABC efflux pump found in epithelial cells of the liver, kidney and intestines, and chemoresistance is secondary to its enhanced efflux activity - translating to poor drug accumulation and suboptimal cytotoxic action (Lohner et al., 2007). In a study of 14 different flavonoids, apigenin was the only small molecule that did not induce P-gp expression in human intestinal Caco-2 cells at the protein level (Lohner et al., 2007). Molecular docking and quantitative structure-activity relationship modeling confirm apigenin as an agent with high P-gp inhibitor binding and potency (Mohana et al., 2016).

Apigenin may have multiple productive interactions with chemotherapeutic agents through its effects on cellular signaling pathways, and this might be of particular importance in cancer cells that have developed further resistance mechanisms (Lefort and Blay, 2011). Apigenin has a synergistic effect with the chemotherapeutic agent 5-FU in inducing apoptosis in p53-mutant human colon adenocarcinoma cells (Zhang et al., 2009). It induces the autophagy mediators LC3-II and Beclin-1 in colorectal carcinoma cells resistant to cisplatin (Chen et al., 2019). Apigenin also enhances the effect of doxorubicin in chemotherapy-resistant hepatocarcinoma cells, likely by down-regulating the PI3K/Akt pathway (Ga et al., 2013). Finally, apigenin is thought to down-regulate the PI3K/Akt pathway and in reduce expression of nuclear factor erythroid 2-related factor (a key player in chemoresistance) at the RNA and protein level (Ga et al., 2013). Combining apigenin with chemotherapeutic drugs may be a worthwhile clinical strategy for the support of cytotoxic action as a chemosensitizer.
